# An Energy-Efficient Clustering Method for Target Tracking Based on Tracking Anchors in Wireless Sensor Networks

**DOI:** 10.3390/s22155675

**Published:** 2022-07-29

**Authors:** Zhiyi Qu, Baoqing Li

**Affiliations:** 1 Science and Technology on Micro-System Laboratory, Shanghai Institute of Microsystem and Information Technology, Chinese Academy of Sciences, Shanghai 201800, China; qzy@mail.sim.ac.cn; 2 University of Chinese Academy of Sciences, Beijing 100049, China

**Keywords:** wireless sensor networks, target tracking, clustering method, energy efficiency

## Abstract

As a key technology in wireless sensor networks (WSNs), target tracking plays an essential role in many applications. To improve energy efficiency, clustering is widely used in tracking to organize the network to achieve data fusion and reduce communication costs. Many existing studies make dynamic adjustments based on static clusters to track moving targets. However, the additional overhead caused by frequent cluster reconstruction and redundant data transmission is rarely considered. To address this issue, we propose a tracking-anchor-based clustering method (TACM) in this paper, in which tracking anchors are introduced to provide activation indications for sensors according to the target position. We use the rough fuzzy C-means (RFCM) algorithm to locate the anchors and use the membership table to activate sensors to form a cluster. Since there are no sending, receiving, and fusing data tasks for anchors, they are lightly burdened and can significantly reduce the frequency of being rotated. Moreover, the state of cluster members (CMs) is scheduled using the linear 0–1 programming to reduce redundant transmissions. The simulation results demonstrate that, compared with some existing clustering methods, the proposed TACM effectively reduces the energy consumption when tracking a moving target, thus prolonging the network lifetime.

## 1. Introduction

Recently, the increased performance of radio and embedded systems and their decreased price have led to the widespread use of Wireless Sensor Networks (WSNs) [1,2,3]. WSNs commonly consist of numerous sensors in the surveillance area, and the sensing unit of a sensor can sense environmental information (e.g., wind speed, temperature, humidity, solar radiation, etc.) [4,5]. The sensing data are transferred to the sink through a wireless communication unit in the sensor. Users can access these sensing data through wireless communication devices, such as terminal hosts, personal computers, smart devices, and laptops.

As one of the critical technologies of WSNs, target tracking is the basis for applications such as military surveillance, wildlife observation, biomedical health care, and vehicle tracking [6,7]. Generally, the main aim of target tracking in WSNs is to detect the existence of the target and to estimate its future position based on historical data of the target movement. In the tracking process, most of the sensors spend a significant amount of time in a state in which they do not participate actively in communications [8]. In this case, we can turn off the radio module of these sensors to save energy. Therefore, how to schedule the state of the sensors in the network to achieve efficient data transmission is a crucial issue for target tracking applications.

**Figure 1:**
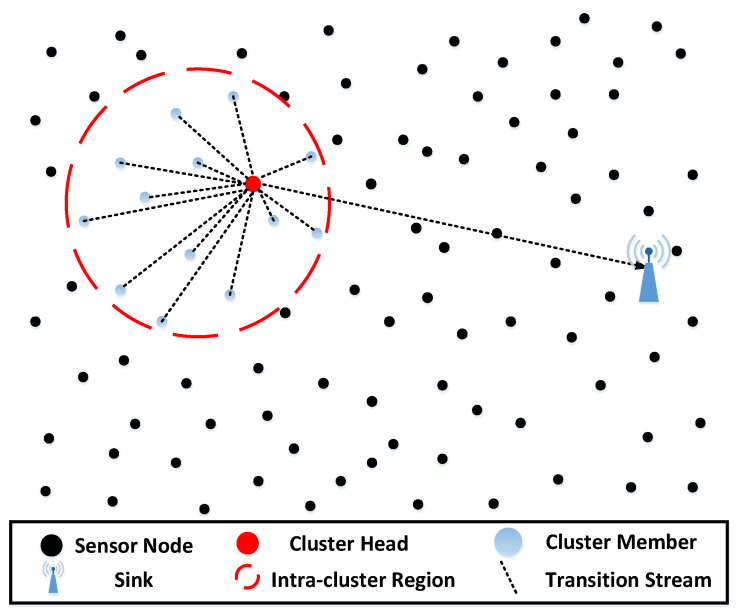
Basic cluster structure in the network.

To deal with this problem, clustering methods are widely used as a means to facilitate cooperative data processing and manage the resources of sensor networks [9,10,11]. As shown in Figure 1, in clustering methods, sensors are organized into clusters, and each cluster has a cluster head (CH) and multiple cluster members (CMs). CMs send sensing data to CH, and CH is responsible for collecting data, performing data fusion, and sending a report toward the sink. In this way, not only can the energy consumption in the network be reduced to achieve the purpose of prolonging the network lifetime, but also the scalability is robust, and the network delay can be reduced.

**Figure 2:**
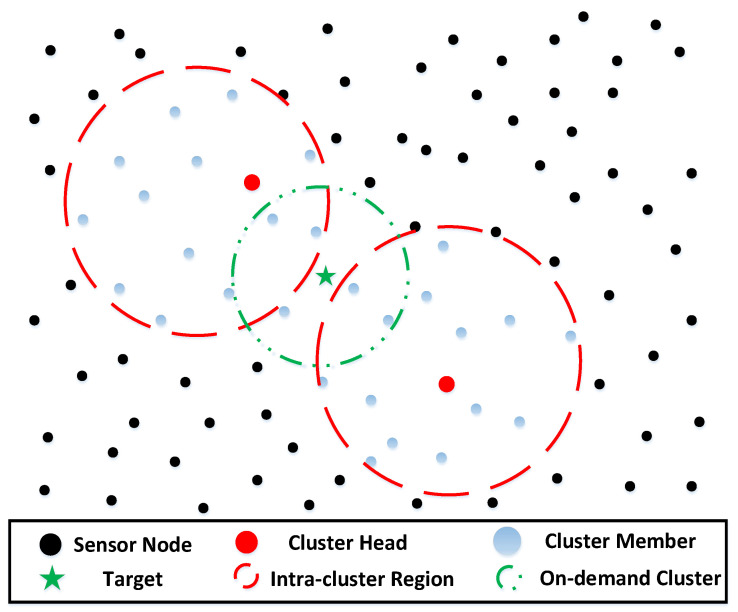
On-demand cluster between static cluster.

Existing studies have designed different clustering methods for target-tracking-based applications, among which the most representative one is forming an on-demand cluster between static clusters [12]. As shown in Figure 2, when the target is located between static clusters, only one on-demand cluster is activated for work, and the data can be transmitted to one CH. The activated cluster is adaptively formed according to the position of the moving target, and the prediction mechanism is used to calculate the target trajectory. One of the CH will fuse the data from CMs in the on-demand cluster to provide the system with a more accurate target position estimate. Although this structure has better energy efficiency than traditional clustering methods, it cannot avoid periodic cluster reconstruction caused by the reselection of CHs. The reason is that the CH not only has to give CMs activation instructions according to the target position, but also undertakes the task of sending, receiving, and fusing data. The death of CH means that the network needs to update the role of the CH in time, and this short-cycle CH rotation will cause additional communication overhead for the system.

To solve the above drawback of existing methods, we propose a novel clustering method for target tracking, in which we abandon the static clustering structure and decentralize the tasks undertaken by CHs. The main objective is to avoid the communication overhead caused by the periodic reconstruction of the cluster structure and to prolong the network lifetime. The major contribution of this paper is summarized as follows:

- Tracking anchors are introduced to indicate sensor activation based on the target position. Since there is no task of sending, receiving, and fusing data, the tracking anchor consumes little energy and therefore does not need to be reselected periodically.
- Using the rough-fuzzy C-means (RFCM) algorithm, we can determine the anchor location, and a membership table will be built according to the sensors’ membership to the lower approximate and boundary region of anchors. The membership table can help the system activate the appropriate sensor set.
- The activated sensor set temporarily forms a dynamic cluster, and the CH is selected employing delay broadcasting. The linear 0–1 programming is used to schedule the state of CMs to reduce the transmission of redundant data in the cluster.

The proposed method generates a dynamic cluster with an energy-efficient topology by decentralizing the tasks of CHs. The uniqueness of the method is that tracking anchors, rather than CHs, are selected in the network. The tracking anchors consume little energy and do not need to be rotated frequently. There will be only one temporarily-formed cluster in the network at each time step, and the CH is also temporarily determined by sensor volunteering. In this way, the tasks of the CH will be undertaken by different sensors so that load balancing can be achieved in the network. Moreover, CMs will be scheduled into appropriate states so that the CH will not collect redundant sensing data.

The remainder of this paper is structured as follows: Section 2 describes some related works. The network model and the energy model are presented in Section 3. Section 4 is dedicated to the presentation of our proposed clustering method. Section 5 provides the simulation results. Finally, Section 6 concludes this paper.

## 2. Related Work

With the rapid development of WSNs, the way of the network organization has attracted the attention of many scholars. However, compared with other typical network structures, the clustering method is a more effective method to reduce the energy consumption of sensor nodes and prolong the network lifetime. In this section, we provide a description of previous works related to clustering methods for target tracking in WSNs.

Wang et al. proposed a hybrid cluster-based target tracking (HCTT) distributed mobility management protocol to solve the boundary problem [12]. By integrating an on-demand cluster into the static cluster structure, HCTT enabled nodes belonging to different clusters share information, thereby ensuring smoothly target tracking and achieving a tradeoff between energy consumption and local sensor collaboration.

In [13], Hu et al. proposed a dynamic clustering method called EEAOC, in which the CH re-adjustment and cluster migration technique were used to activate appropriate clusters for continuous event monitoring via a 2-logical-overlapping clustering scheme. In EEAOC, the cluster topology can be switched according to the target movement with low communication overhead.

The authors in [14] proposed a dynamic clustering protocol (EEDC) for target tracking and continuous event monitoring. In EEDC, the overlapping cluster structure was dynamically reconstructed to adapt to the changing location of targets, and candidate cluster heads were selected according to rough fuzzy C-Means (RFCM) and genetic algorithm (GA). EEDC can significantly reduce system energy consumption as compared to other clustering protocols.

To provide a good tradeoff between energy efficiency and tracking accuracy, a convolution-based method was presented to quantify the relationship between the cluster parameters and the energy-quality metrics of the tracking system in [15]. The proposed method can provide Pareto-optimal parameters to obtain the optimal energy efficiency of a cluster-based WSN tracking system.

In [16], Feng et al. proposed a dynamic chain-based sensor collaboration (DCBC) method for target tracking, which has a good reference value for clustering methods. Compared to clustering, chain structures involve a large number of unnecessary nodes and unacceptable transmission delays. Therefore, in DCBC, the authors dynamically adjusted the network topology by pruning and adding nodes instead of re-establishing the chain structure. The experimental results showed that DCBC can reduce and balance the average energy consumption of the network.

Ahmad et al. proposed an energy-efficient adaptive clustering scheme (EEAC) for target tracking with adaptive cluster size to keep minimal energy consumption while obtaining high tracking accuracy [17]. In EEAC, the authors presented a model for the prediction of target location and described how adaptive clusters were formed and how CHs were selected. It was observed that EEAC required less energy consumption as compared to other approaches.

In [18], aiming to solve the problem of collaborative tracking, Arienzo et al. laid out a cluster-based architecture to address the limitations in computational power, battery capacity, and communication capacities of sensors. Target tracking was formulated as a cross-layer optimization problem for the purpose of maximizing the utility function in the cluster. Experiments indicated that the proposed architecture can reduce the energy consumption caused by node selection in a dynamic scenario.

Wang et al. proposed a hierarchical clustering node collaborative scheduling (HCNCS) protocol in [11]. In HCNCS, nodes were classified into sensing disks for sensing and transmitting data. Node rotation was used to balance energy consumption, and cooperative sensing can improve the coverage area of sensing disks. In this way, the coverage performance of the network can be improved, and energy consumption can be balanced.

Using state prediction technology and the Particle Swarm Optimizer (PSO), Bhagat designed a tracking mechanism to achieve coordination between nodes and continuous target tracking in [19]. The author used state prediction technology to calculate the target trajectories and used PSO to optimize the network topology during tracking to save overall overhead. The experimental results indicated that the proposed method has good performance in energy management and can effectively prolong the network lifetime.

In [20], a fault-tolerant sensor scheduling (FTSS) approach was proposed to reduce the target loss probability for target tracking in WSNs. In FTSS, the fault-tolerant domain was introduced, and the binary Gray Wolf Optimizer (bGWO) was used to activate the optimal tracking node set. Experiments indicated that reducing the target loss probability by FTSS can not only improve the tracking accuracy but also save the extra energy consumption brought by the recovery mechanism.

A dynamic cluster task allocation scheme is proposed in [21] to perform collaborative task allocation for target tracking in WSNs. By considering energy consumption and residual energy balance, an energy efficient model was proposed. Moreover, a tracking accuracy model based on the area-sum principle was presented using triangulation localization. The two models were combined to establish a CM selection method based on the genetic algorithm (GA) for multi-target tracking. Simulation results indicated the high energy efficiency of the model and the validity of GA in CM selection.

In [22], Fu et al. proposed an improved algorithm, LEACH-MTC, which was based on the LEACH protocol. First, using the extended Kalman filter (EKF), the future location of the moving target can be obtained. After that, combining the state prediction of the target and the performance of collaborative monitoring, an elliptical monitoring area was constructed to align with the moving direction of the target. It was shown that LEACH-MTC could increase the survival number of working nodes and reduce network energy consumption.

Soderlund et al. proposed a rapid and efficient clustering method in [23]. The main aim is to achieve optimal sensor allocation for localization uncertainty reduction in target tracking. By maximizing the expected posterior of the moving target, the derived cluster was used as the search space for optimal sensor allocation. The proposed method was indicated to be superior in terms of achieving greater uncertainty reduction.

Considering failures happening in target tracking applications, a novel fault-tolerant target tracking (FTTT) protocol based on clustering was proposed in [9]. In FTTT, a static clustering method was presented, which can facilitate the CH recovery procedure. Moreover, redundant nodes were scheduled into a sleep state to reduce the cost of fault tolerance. FTTT has been proven to effectively recover faulty nodes and reduce the total energy consumption in the network.

In [24], Liao et al. proposed a clustering method for target tracking with distributed information compression called CTCI, in which a novel cooperative positioning approach was developed to keep the information of measurement uncertainties. Moreover, CTCI allowed the active nodes to use information compression for energy conservation. The Bayesian framework was utilized to provide a complete distribution of target position estimation. Simulation results indicated that CTCI achieved a balanced performance in terms of tracking accuracy, transmission data size, and energy consumption.

In general, almost all the clustering methods for target tracking focus on solving the energy consumption issue. Currently, making dynamic adjustments to the classical static cluster structure is the most effective way to track moving objects. CHs need to schedule their CMs to perform operations such as cluster migration and on-demand cluster formation according to the target position. However, in this way, the workload on the CHs will be heavy, which may cause their energy to be quickly exhausted. To maintain the structure, frequent CH rotation and cluster formation cannot be avoided in the network, resulting in additional communication overhead. This is a common problem of existing clustering methods which has been rarely paid attention to. Therefore, for the purpose of improving energy efficiency, we hope to propose a novel clustering method in this paper to decentralize the workload of CH and reduce the communication overhead caused by frequent rotation.

## 3. System Model

### 3.1. Network Model

In this paper, the network is composed of numerous sensors as well as a sink node. N sensor nodes are randomly deployed in a M×M monitoring area, and the sink is placed in the center. The deployed sensor nodes are homogenous, and all have the same capabilities and limited power source, of the same initial energy, that cannot be recharged or replaced. Assume that the sink has no energy constraints. We make some basic assumptions as follows [25,26]:

1. All the sensor nodes are static and do not change their location once deployed.
2. Each node can be identified by its unique ID that differs from other nodes.
3. All the sensors have knowledge of their location according to an equipped GPS.
4. The collisions during transmission are not considered in the network, and the radio channels are symmetric.
5. The sensing radius and communication radius of each node are $r_{s}$ and $r_{c}$, respectively. Set $r_{c}\;\geq \;2\;r_{s}$ to ensure that all nodes that sense the target can communicate with each other.
6. The sensing model of the node adopts the Boolean omnidirectional model, which is given by [27]:
  (1) $$P(s_{j},\;tar)=\left\{ \begin{array}{l} 1,\;\;\;\;\;\;\;\;\;\;d_{s_{j},tar}\leq r_{S}\\ 0,\;\;\;\;\;\;\;\;\;\;d_{s_{j},tar}>r_{S} \end{array}\right.\;,$$
  where $P(s_{j},\;tar)$ is the probability that sensor $s_{j}$ can sense the target, and $d_{s_{j},tar}$ is the distance from sensor $s_{j}$ to target. $r_{s}$ is the sensing radius.

### 3.2. Energy Model

**Figure 3:**
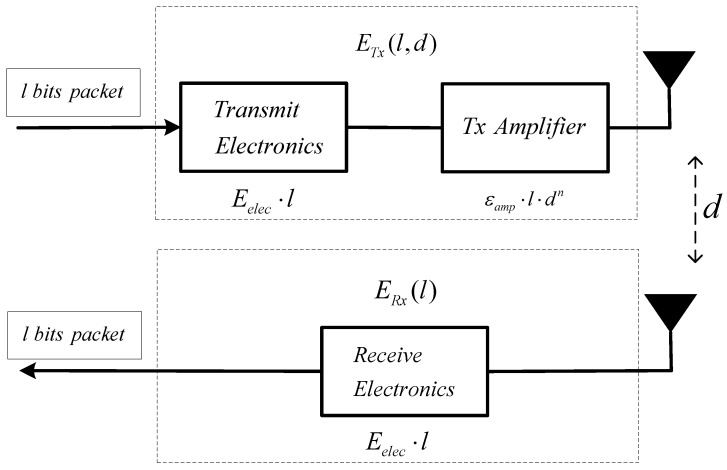
Energy model.

We adopt the same radio model applied in [14,25,28] to calculate the energy consumption. As shown in Figure 3, the energy consumption for transmission contains two parts: transmitter and receiver. Once the signal is generated by the transmitter, the amplifier will strengthen it using two different powers according to the transmission distance. Therefore, the energy model for transmission is divided into the free space model for short-haul communication and the multipath fading model for long-distance communication.

The energy $E_{Tx}$ used for each node to transmit an l bits package over communication distance of d is given as follows [14]:

(2) $$E_{Tx}(l,d)=\left\{ \begin{array}{l} l\cdot E_{elec}+l\cdot \varepsilon_{fs}\cdot d^{2},\;\;\;if\;\;\;d<d_{0}\\ l\cdot E_{elec}+l\cdot \varepsilon_{mp}\cdot d^{4},\;\;if\;\;\;d\geq d_{0} \end{array}\right.$$

where $E_{elec}$ denotes the energy consumption to run the transmitter or receiver circuit, $\varepsilon_{fs}$ and $\varepsilon_{mp}$ denote the amplification coefficient for the free space model and the multi-path fading model, respectively, and $d_{0}$ is a threshold distance calculated by [28]:

(3) $$d_{0}=\sqrt{\frac{\varepsilon_{fs}}{\varepsilon_{mp}}}\;.$$

The energy $E_{Rx}$ used for receiving an l bits package can be calculated by [14]:

(4) $$E_{Rx}(l)=l\cdot E_{elec}\;.$$

## 4. The Proposed Tracking Anchor Based Clustering Method

In this section, a detailed illustration of a tracking-anchor-based clustering method (TACM) will be given. TACM contains four phases: tracking anchor selection, node activation, cluster formation, and state scheduling for CMs. First, the rough-fuzzy C-means (RFCM) algorithm is used to determine the location of the tracking anchor and the affiliation of surrounding nodes. Then the membership table is built, and the rules for node activation are proposed. After that, the activated nodes become the tracking node set, and the cluster head (CH) is selected by the delay broadcast mechanism. Finally, using linear 0–1 programming, CMs are scheduled to the appropriate state to reduce redundant transmissions.

### 4.1. Tracking Anchor Determination

After being deployed to the surveillance area, sensor nodes remain in the sleep state. To activate a set of tracking nodes in advance, a certain number of tracking anchors are selected in the network. Tracking anchors are responsible for sending scheduling instructions to nearby nodes according to the predicted target position.

First, we determine the number of required tracking anchors. Considering that the sum of the sensing coverage of all anchors is not less than the surveillance area, we can obtain:

(5) $$K\pi r_{S}^{2}\geq M\times M\;,$$

where K denotes the number of tracking anchors. For energy efficiency, we select as few anchors as possible while the coverage condition is satisfied. K is given by:

(6) $$K=\left\lceil \frac{M\times M}{\pi r_{S}^{2}}\right\rceil \;.$$

After determining the number of anchors, the RFCM algorithm proposed in [29] is used to perform unsupervised clustering of sensor nodes. K cluster centers obtained after iteration can be designated as the position of tracking anchors in the network. In RFCM, N nodes are partitioned into K clusters by minimizing the objective function $J_{RF}$:

(7) $$J_{RF}=\left\{ \begin{array}{l} \omega_{1}\times A+\omega_{2}\times B,\;\;\;if\;\underline{A}(\beta_{i})\neq \emptyset ,\;B(\beta_{i})\neq \emptyset \\ \;\;\;\;\;\;\;\;\;\;A,\;\;\;\;\;\;\;\;\;\;\;\;\;\;\;\;if\;\underline{A}(\beta_{i})\neq \emptyset ,\;B(\beta_{i})=\emptyset \\ \;\;\;\;\;\;\;\;\;\;B,\;\;\;\;\;\;\;\;\;\;\;\;\;\;\;\;if\;\underline{A}(\beta_{i})=\emptyset ,\;B(\beta_{i})\neq \emptyset \end{array}\right.\;,$$

(8) $$A=\sum_{i=1}^{K}\sum_{s_{j}\in \underline{A}(a_{i})}{\left(\mu_{ij}\right)}^{m}{\left\|s_{j}-a_{i}\right\|}^{2}\;,$$

(9) $$B=\sum_{i=1}^{K}{\sum_{s_{j}\in B(a_{i})}{\left(\mu_{ij}\right)}^{m}\left\|s_{j}-a_{i}\right\|}^{2}\;,$$

where $\beta_{i}$ denotes the cluster with the anchor $a_{i}$ as the center; $\underline{A}(\beta_{i})$ and $B(\beta_{i})$ denote the lower approximation region and boundary region of cluster $\beta_{i}$, respectively; parameters $\omega_{1}$ and $\omega_{2}$ correspond to the relative importance of the lower and boundary regions, and $\omega_{1}+\omega_{2}=1$; m is the fuzzifier, where 1≤m<∞, which is introduced to calculate the relevance of each node to the anchors, rather than assigning it to the nearest anchor. If the value of m is too large, good clustering cannot be obtained, and if m is too small, the algorithm is close to the normal hard clustering C-means algorithm; $\mu_{ij}$ is the probabilistic membership of the sensor $s_{j}$ to anchor $a_{i}$, and $\mu_{ij}\in [0,1]$; ‖⋅‖ denotes the distance norm.

**Figure 4:**
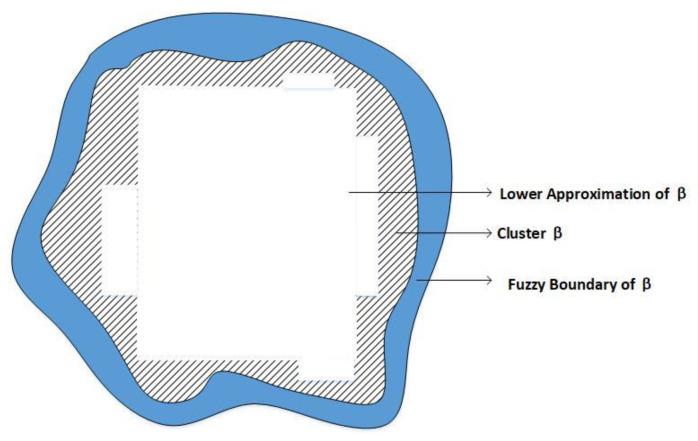
Cluster β and its lower approximation region and fuzzy boundary region.

In the applied RFCM, each cluster consists of a clear lower approximation region and a fuzzy boundary region, as shown in Figure 4. The tracking anchor is located at the centroid of the cluster. There are two important properties about $\underline{A}(\beta_{i})$ and $B(\beta_{i})$:

***Property I:***

If the sensor $s_{j}\in \underline{A}(\beta_{i})$, then $x_{j}\notin \underline{A}(\beta_{k}),\forall k\neq i$. That is, the sensor $s_{j}$ is contained in $\beta_{i}$ definitely.

***Property II:***

If the sensor $s_{j}\in B(\beta_{i})$, then $s_{j}$ possibly belongs to $\beta_{i}$ and potentially belongs to another cluster.

Sensors located in the lower approximation region and the boundary region have different influences on the location of anchors. In RFCM, the membership value of sensors in the lower approximation is fixed to 1, that is, $\mu_{ij}=1$ if $s_{j}\in \underline{A}(\beta_{i})$. Moreover, the membership value of the sensors in the boundary region is calculated according to the relative distance to the anchor:

(10) $$\mu_{ij}={\left(\sum_{k=1}^{K}{\left(\frac{d_{ij}}{d_{kj}}\right)}^{\frac{2}{m-1}}\right)}^{-1},\;\;\;if\;s_{j}\in B(\beta_{i})\;,$$

where $d_{ij}$ and $d_{kj}$ denote the distance from sensor $s_{j}$ to anchor $a_{i}$ and $a_{k}$, respectively. The new position of the anchor is calculated based on the weighted average of the lower approximation and boundary, which is given by Equation (11):

(11) $$a_{i}^{RF}=\left\{ \begin{array}{l} \lambda_{1}\times ℭ+\lambda_{2}\times D,\;\;\;if\underline{A}(\beta_{i})\neq \emptyset ,\;B(\beta_{i})\neq \emptyset \\ \;\;\;\;\;\;\;\;\;\;\;\;ℭ,\;\;\;\;\;\;\;\;\;\;\;\;\;\;\;if\underline{A}(\beta_{i})\neq \emptyset ,\;\;B(\beta_{i})=\emptyset \\ \;\;\;\;\;\;\;\;\;\;\;\;D,\;\;\;\;\;\;\;\;\;\;\;\;\;\;if\underline{A}(\beta_{i})=\emptyset ,\;\;B(\beta_{i})\neq \emptyset \end{array}\right.\;,$$

(12) $$ℭ=\frac{1}{\left|\underline{A}(\beta_{i})\right|}\sum_{x_{j}\in \underline{A}(\beta_{i})}s_{j}\;,$$

(13) $$D=\frac{1}{n_{i}}\sum_{s_{j}\in B(\beta_{i})}{\left(\mu_{ij}\right)}^{m}s_{j}\;,$$

where

(14) $$n_{i}=\sum_{s_{j}\in B(\beta_{i})}{\left(\mu_{ij}\right)}^{m}\;,$$

where $\left|\underline{A}(\beta_{i})\right|$ denotes the number of sensors in the lower approximation region. The position of the anchor depends on the parameter $\lambda_{1}$ and $\lambda_{2}$, and the fuzzifier m rules their relative influence. Since sensors in the lower approximation region definitely belong to a certain anchor, they are assigned a higher weight $\lambda_{1}$ compared to $\lambda_{2}$ of the sensors in the boundary region. Thus, the values are given by $0<\lambda_{2}<\lambda_{1}<1$.

The optimization process of RFCM to the objective function $J_{RF}$ is calculated by Equation (11). Firstly, randomly assign K positions to anchors to form an initial population $A=\{a_{1},\;\;a_{2},\;\ldots ,\;a_{{}_{K}}\}$, and the fuzzy membership values of sensors are calculated according to (10). These anchors and sensors constitute the initial population. Then, we sort the membership value of each sensor and obtain the highest value, which is assumed to be $\mu_{ij}$, and the second-highest value, which is assumed to be $\mu_{kj}$. If $(\mu_{ij}-\mu_{kj})\leq \delta$, we have $s_{j}\in B(\beta_{i}),\;\;s_{j}\in B(\beta_{k})$, and the sensor $s_{j}$ does not belong to the lower approximation region of any anchor. Otherwise, if $(\mu_{ij}-\mu_{kj})>\delta$, then $s_{j}\in \underline{A}(\beta_{i})$. The performance of RFCM depends on the value of δ, which can be defined by Equation (15):

(15) $$\delta =\frac{1}{N}\sum_{j=1}^{N}\left(\mu_{ij}-\mu_{kj}\right)\;.$$

where N is the total number of sensors, and $\mu_{ij}$ and $\mu_{kj}$ are the highest and second-highest membership of the sensor $s_{j}$, respectively. The value of δ represents the average difference between the two highest memberships value of all the nodes in the network, which is dependent on the location of the anchors and the nodes. After determining the region to which the sensor belongs, the anchor’s position is updated according to Equation (11).

The above steps are repeated until the maximum number of iterations $I_{max}$ is reached or the anchor position and membership relationship obtained by the two iterations are stable, that is $\left|\frac{\mu_{ij}(t)-\mu_{ij}(t-1)}{\mu_{ij}(t-1)}\right|<\varepsilon$. Both $I_{max}$ and ε are used as signs to stop the iteration. $I_{max}$ can be obtained from the empirical convergence iterations of the RFCM algorithm in the experiment. ε is the threshold of the change rate of the membership value for two iterations, generally taking a small value, e.g., ε=0.01. After obtaining the anchor position and the membership relationship of sensors, there are two ways to deploy the anchor: if the environment allows, we can use vehicles, UAVs, etc. to transport additional sensors to the designated position as anchors. Otherwise, we make the sensor closest to the anchor position be the anchor.

The algorithm for tracking anchor determination based on RFCM is shown below (Algorithm 1):
**Algorithm 1.** Tracking Anchor Determination Based on RFCM**Input:** Node Number N, Tracking Anchor Number K **    Output:** Tracking Anchor Locations, Node Membership    1: Parameter Initialization: $I_{max}$, Fuzzifier m, Thresholds δ and ε
    2: Initialize Population $A=\{a_{1},\ldots ,\;\;a_{i},\ldots ,\;\;a_{{}_{K}}\}$    3: **Repeat**    4:     **For** Each $a_{i}$ in A **do**    5:       Calculate $\mu_{ij}$ for K anchors and N nodes using Equation (10)6:     **End for**    7:     **If**
$\mu_{ij}$ and $\mu_{kj}$ be the two highest membership of $s_{j}$ and $\left|\mu_{ij}-\mu_{kj}\right|\leq \delta$   **then**    8:       $s_{j}\in \bar{A}(\beta_{i})$ and $s_{j}\in \bar{A}(\beta_{k})$
    9:     **Else**    10:      $s_{j}\in \underline{A}(\beta_{i})$     11:    **End if**    12:    Modify $\mu_{ij}$ considering lower and boundary regions    13:    Compute new anchors as Equation (11)    14: **Until** $I=I_{max}$ or |μ(t)−μ(t−1)|<ε    15: **For** each sensor in the network **do**    16:    Assigned as a member to the anchors with maximum μ17: **End for**

### 4.2. Node Activation

Activation of nodes requires scheduling instructions provided by anchors. By using RFCM, we obtain tracking anchors, along with their lower approximation regions and boundary regions. According to the membership relationship, we build a membership table of the anchor for common sensors. The membership table has N rows and K columns, which refer to N sensors and K anchors, respectively. The value of the j-th row and the i-th column of the member table is MT(j,i), where MT(j,i) is equal to 0 or 1, indicates that the sensor $s_{j}$ belongs to $a_{i}$’s lower approximation region or boundary region. Furthermore, MT(j,i)= 0 indicates that $s_{j}$ is far away from $a_{i}$ without any membership relationship.

According to the properties mentioned in Section 4.1, sensors can be divided into two types: one belongs to the lower approximation region of an anchor. Since they do not belong to any other anchors, they only have one ‘1′ in their rows on the membership table. Another belongs to the boundary regions of multiple anchors, apparently with multiple occurrences of ‘1’ in its row.

**Figure 5:**
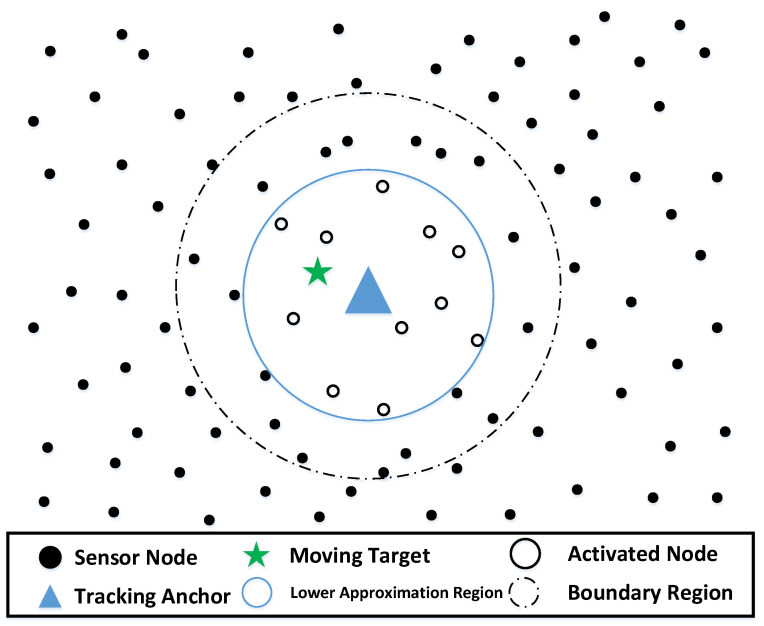
Sensor Activation Scenario (1).

**Figure 6:**
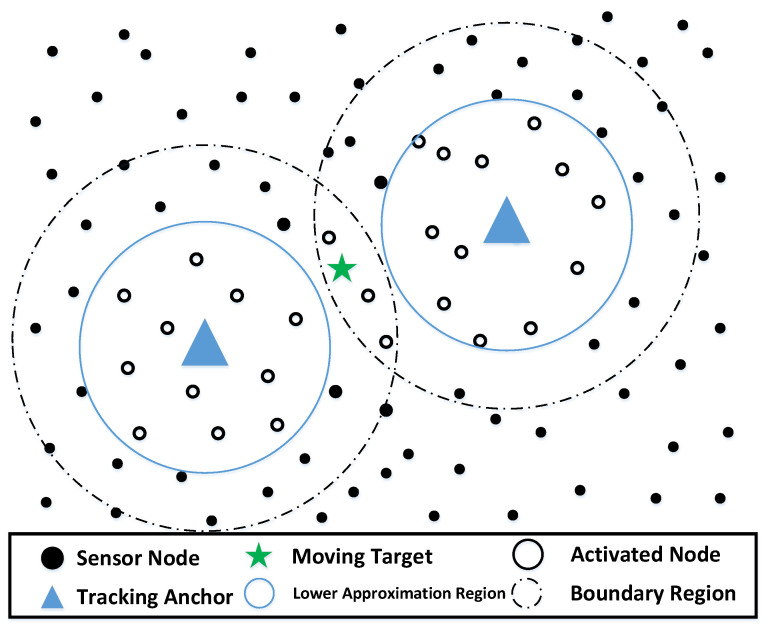
Sensor Activation Scenario (2).

The activation rules for the sensors are given with the help of the membership table: A sensor will be activated when it can receive the “Node Activation” messages sent by all the anchors corresponding to the column with the value “1” in its row. Following this rule, we specifically analyze two sensor scheduling scenarios in the tracking process:

(1) The target is located in the lower approximation region of a certain anchor. As shown in Figure 5, only the sensors in the lower approximate region will be activated because they have received a “Node Activation” message from the only anchor to which they belong.

(2) The target is located in the overlapping boundary region of multiple anchors. As shown in Figure 6, the nodes in the lower approximation regions and the overlapping boundary region of the two anchors will be activated. The overlapping boundary region of the two anchors can be used as a transition region to ensure the continuity of the tracking process.

### 4.3. Cluster Formation

We consider the future-position estimation mechanism as technical support, which is commonly used by traditional dynamic clustering algorithms [8]. As the target moves in the surveillance area, the tracking anchors near the predicted target position will send activation messages to sensors. The activated sensors will form a cluster. The CH will be selected among them using a delayed broadcast message.

Assuming that the target’s next position is predicted to belong to the scheduling region of some anchors, the sink will send an “Anchor Scheduling” message to them. Note that this message is relayed by other anchors in the network because most sensors are sleeping. The anchor receiving the message will broadcast a “Node Activation” message to the nodes in its lower approximation region and boundary region, as described in Section 4.2. Activated nodes will broadcast a “Cluster Formation” message by themselves after waiting for a while. The sensor that broadcasts the message first becomes the CH, and the sensor that receives the message becomes the CM. The length of delay time is related to the distance to the target’s predicted position and the residual energy of the sensor, which can be given by:

(16) $$t_{delay}=T_{\min}+(T_{\max}-T_{\min})*(\eta \frac{d}{e})+T_{rand}\;,$$

where $T_{\min}$ and $T_{\max}$ denote the minimal and maximum delay timer values; d is the distance from a sensor to the target; e denotes the residual energy; η is a parameter that can nondimensionalize distance and energy and $T_{rand}$ is a random delay timer value, which is set to prevent multiple sensors from becoming CHs at the same time.

In each round, the CMs send sensing data to CH. Then CH will fuse the data and forward them to the sink. When the target moves out of the current cluster, the system will activate the sensors to form the next tracking cluster in advance to ensure continuous tracking of the target. This process continues until the target moves out of the monitoring range or the network dies.

### 4.4. State Scheduling for CMs

To improve the effectiveness of the network, we further schedule the working state of the CMs in the current tracking cluster. During the tracking process, it is not necessary for all CMs to send sensing data to CH. Therefore, we can find ways to reduce the energy consumption caused by redundant data transmission. We first set the activated CMs to have two working states: sensing state and sensing-transmitting state. The sensor in the sensing state only has the sensing module turned on and will not send data to the CH. On the other hand, the sensor in the sensing-transmitting state will sense and transmit data to the CH. By setting redundant nodes to be in the sensing state, we can reduce energy consumption and extend network life.

Considering tracking quality and node coverage redundancy, we transform the problem of determining the optimal working status of CMs into a linear 0-1 programming problem. We define the variable x to represent the two working states of the CMs:

(17) $$x=\left\{ \begin{array}{l} 0,\;\;\;\;sensor\;works\;\;in\;\;sensing\;\;state\;\;\\ 1,\;\;\;\;sensor\;\;works\;\;in\;\;sensing-transmitting\;\;state\; \end{array}\right.\;.$$

Apparently, the CMs working in the sensing-transmitting state will consume more energy than the CMs working in the sensing state because of the data transmission. To reduce and balance energy consumption, we need to schedule as few CMs as possible to work in the sensing-transmitting state and ensure that they are the ones with more energy remaining. Therefore, the objective function F(x) is defined as:

(18) $$\;F(x)=\sum_{j=1}^{n}e_{j}x_{j}\;,$$

where n is the number of the CMs in the cluster, and $e_{j}$ is the energy currently consumed by sensor $s_{j}$. We can achieve the purpose of reducing and balancing energy consumption by minimizing F(x).

Then there are the constraints. First, it is necessary to ensure that the data transmitted by the CMs meet the requirements of tracking quality, so the number of CMs in the sensing-transmitting state should be greater than or equal to the minimum number required in specific tracking applications, which is defined as $n_{0}$. The constraint is given by:

(19) $$\sum_{j=1}^{n}x_{j}\geq n_{0}\;.$$

Moreover, in a large-scale network with randomly deployed nodes, the sensing coverage of some nodes will overlap with each other. When the target sensing source is generated in the overlapped part, multiple nodes will sense the same signal, and several identical data packets will be transmitted to CH, resulting in data redundancy. There will also be competition between nodes for the same communication channel, resulting in unnecessary energy loss. Therefore, we set the second constraint to ensure that the CMs in the sensing-transmitting state are not redundant nodes according to the center angle of the overlapping coverage area being less than 2π, that is, $\phi (x_{j})\leq 2\pi$, so the constraint is given by:

(20) $$\;x_{j}\cdot \phi (x_{j})\leq 2\pi \;.$$

Regarding $\phi (x_{j})$, according to Equation (20), the calculation is meaningful only when the node $s_{j}$ is in the sensing-transmitting state, that is $x_{j}=1$. To obtain $\phi (x_{j})$, we first set the sensing area of node $s_{j}$ as $A_{j}$. The neighbors of $s_{j}$ are defined as other CMs that are in the sensing-transmitting state: $N(x_{j})=\{s_{k}\in cluster\;|\;x_{k}=1,\;k\neq j\}$. If $s_{j}$ is a redundant node, it needs to satisfy [30]:

(21) $$A_{j}\subseteq \underset{s_{k}\in N(s_{j})}{\cup }\left(A_{k}\cap A_{j}\right)\;.$$

**Figure 7:**
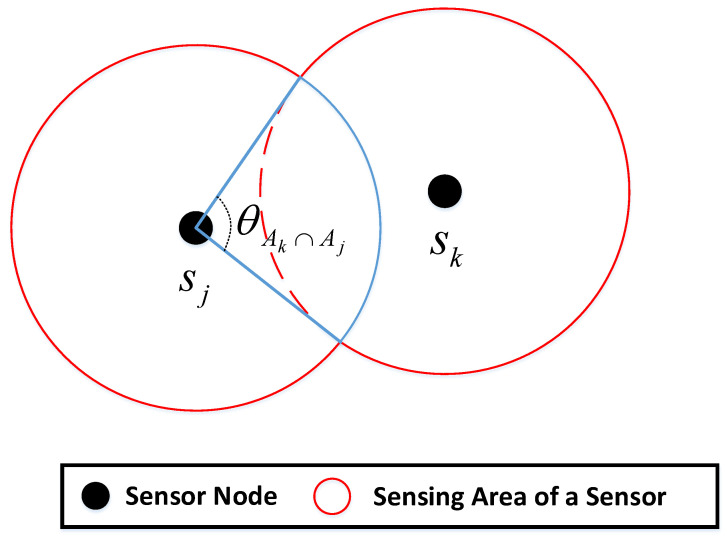
The overlap of the sensing area of two nodes.

The overlap of two nodes’ sensing areas can be simplified as a sector, which can be represented by a central angle, as shown in Figure 7. Therefore, calculating the overlapping coverage area of a node can be equivalent to calculating the sum of the central angles of the neighbors covering the node, which is given by:

(22) $$\phi (x_{j})=\underset{s_{k}\in N(s_{j})}{\cup }\theta_{A_{k}\cap A_{j}}\;,$$

According to Equations (17)–(20), we can define the linear 0–1 programming problem to be solved as:

(23) $$min\;\;F(x)=\sum_{j=1}^{n}e_{j}x_{j}$$

(24) $$s.t.\left\{ \begin{array}{l} \sum_{j=1}^{n}x_{j}\geq n_{0}\\ \;x_{j}\cdot \phi (x_{j})\leq 2\pi \\ \;x_{j}=0\;\;or\;\;1 \end{array}\right.\;.$$

To solve this node state scheduling problem, we adopt the implicit enumeration method [31]. First, we find a feasible solution tentatively, that is, by selecting q CMs to be in the sensing-transmitting state, satisfying the constraints. Then we calculate the objective value of this feasible solution. Assuming F=Q is calculated, we will add it to the constraints as a filtering constraint, and (24) will become:

(25) $$s.t.\left\{ \begin{array}{l} \sum_{j=1}^{n}x_{j}\geq n_{0}\\ \;x_{j}\cdot \phi (x_{j})\leq 2\pi \\ \sum_{h=1}^{q}e_{h}x_{h}\leq Q\\ \;x_{j}=0\;\;or\;\;1 \end{array}\right.\;.$$

In this way, we have four constraints and enumerate all the $2^{n}$ solutions and filter out infeasible solutions using the constraints in (25). For each solution, we substitute it into each constraint to see if it satisfies the inequalities. If one constraint is not satisfied, the other constraints do not have to be checked. Due to the increased constraint, the number of computations can be reduced. During the calculation, if the objective value of a particular solution is U, and U<Q, then we replace the third constraint in (25) with $\sum_{h=1}^{q}e_{h}x_{h}\leq U$. This improvement of filter conditions can further reduce the amount of calculation. The optimal solution can be obtained by iterating the above calculation until the value of F cannot be smaller. The optimal solution denotes the optimal status of the CMs in the cluster.

The intra-cluster state scheduling process is described in Algorithm 2.
**Algorithm 2** State Scheduling for CMs**Input:** Cluster Member Number n, node in the sensing or sensing-transmitting state x=0 or 1
 **Output:** Optimal State of Cluster Members 1: **Parameter Initialization:** Consumed Energy e, Minimum Number of Nodes to Transmit $n_{0}$
 2: **Initial Solution:** Randomly selecting q CMs to be in the sensing-transmitting state, satisfying Equation (24), and calculate the objective function value as Q using Equation (23). 3: Add F≤Q to the constraints, Equation (24) becomes Equation (25) 4: Enumerate $2^{n}$ solutions.  5: **For** each solution 6:     **If** Equation (25) is satisfied **then** 7:       Calculate the objective value by Equation (23) to be U 8:       **If** U<Q **then** 9:           Set Q=U in the third constraint of Equation (25)  10:   **Else**
 11:       The solution is eliminated.12: **End for** 13: The value of Q cannot be smaller, and the optimal solution can be obtained, which denotes the optimal status of CMs.

**Figure 8:**
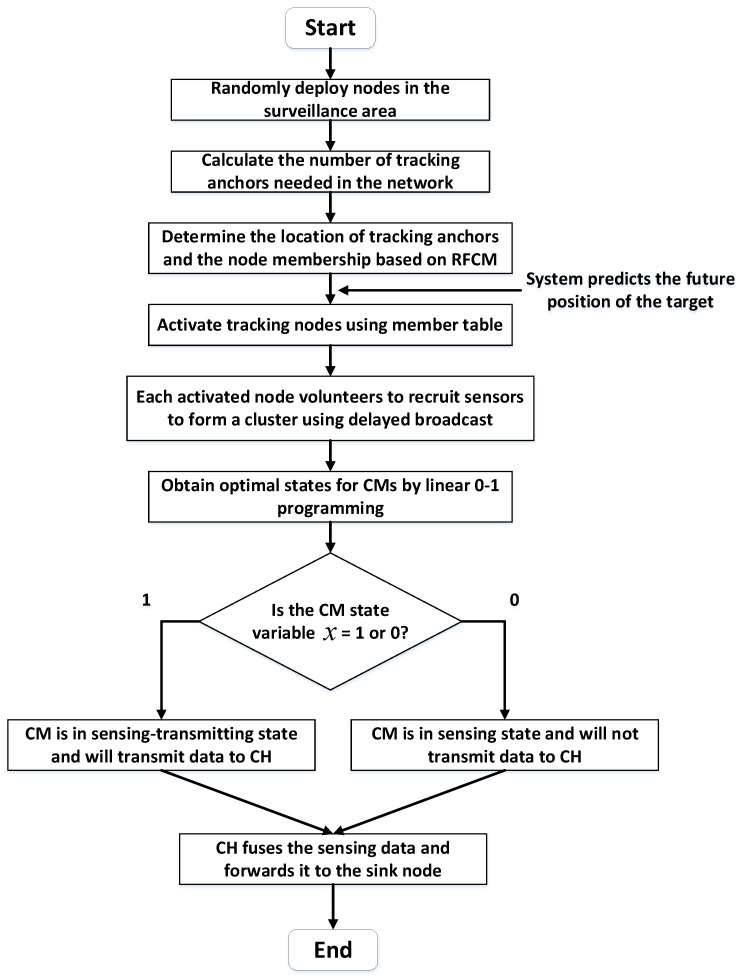
Workflow of the proposed algorithm.

Overall, the proposed algorithm works as follows: First, using RFCM, the position of the tracking anchors and the memberships of nodes are determined. Instead of taking on the task of sending, receiving, and fusing data, the tracking anchors are only responsible for broadcasting activation messages to nodes in their lower approximation and boundary regions when a target is predicted to move nearby. Then, we will maintain a membership table, in which we record each node’s membership to the anchors. According to the information provided by the membership table, a node will only be activated when it has received activation messages from all anchors to which it belongs. After that, the nodes activated by the tracking anchors will form a dynamic cluster. The CH is selected using a delayed broadcast related to distance and energy. To reduce redundant data transmission, we design a sensing state and sensing-transmitting state for CMs and use linear 0–1 programming to select the appropriate working states for CMs. Finally, the CMs in the sensing-transmitting state will send data to the CH, and the CH will fuse the data and forward them to the sink. The workflow of the proposed algorithm is shown in Figure 8.

In the proposed algorithm, tracking anchors play a crucial role in indicating the activation of tracking nodes, which can be used as a substitute for traditional static CHs. Since there is no heavy transmission task like the static CHs, the rotation period of the tracking anchors can be longer. Thus, the communication overhead caused by cluster structure reconstruction can be saved. Moreover, after the dynamic cluster is formed, we set two working states for the CMs and do not require all CMs to transmit their sensing data. In the scheduling process, we try to avoid redundant transmission within the cluster while meeting the tracking quality requirements. As a result, the proposed algorithm can further save energy and extend network lifetime.

## 5. Performance Evaluation

In this section, we evaluate the performance of the proposed algorithm in the WSN, and the existing HCTT [12], EEAOC [13], and EEDC [14] algorithms are used in comparison in the simulation experiments. In HCTT, static clusters and on-demand dynamic clusters alternately perform the tracking tasks. By scheduling boundary nodes to form an on-demand cluster, HCTT can solve the problem of limited sensor information sharing between static clusters. EEAOC is a clustering algorithm with a 2-logical cluster structure. The affiliation between nodes and different CHs will change according to the movement of the target. In EEDC, a dynamic cluster capable of omnidirectional tracking is constructed to cope with the random movement of the target. RFCM and GA algorithms are used to select on-demand candidate CHs. In general, the above three comparison algorithms are all energy efficient clustering protocols proposed for target tracking applications in WSNs.

### 5.1. Simulation Setup

Simulation experiments were carried out using MATLAB 2016a (MathWorks, Natick, MA, USA). For the convenience of comparison, we refer to [12,13,14] to make a comprehensive experiment parameter setting. The parameters that appeared in simulations are listed in Table 1. A wireless sensor network consisting of 200–500 homogeneous sensor nodes randomly distributed in 500 m × 500 m square area with identical initial energies is considered. The sink is placed at the coordinate (250, 250). The sensing range and communication range of each sensor are identically fixed to 20 m and 40 m. The size of the data packet is 4000 bits. For the determination of the tracking anchors, the maximum iteration is 300, and we terminate the iteration when $\left|\frac{\mu (t)-\mu (t-1)}{\mu (t-1)}\right|<\varepsilon$. The quantity ε is a small value, controlling the convergence of the membership μ. In our experiments, ε=0.01, that is, our results obtained converged to the 0.01 specification level for comparing the two different methods [32,33]. The simulation lasted for 5000 time steps.

As for the target, we simulate a scenario where the target moves based on a Gauss–Markov mobility model [34] in the surveillance area. Initially, a random speed and direction are assigned to each target. At each time step t, the movement parameters of the target are updated by:

(26) $$v_{t}=\eta v_{t-1}+(1-\eta^{2})\bar{v}+\sqrt{1-\eta^{2}}v_{t-1}^{G}\;,$$

(27) $$\theta_{t}=\eta \theta_{t-1}+(1-\eta )\bar{\theta }+\sqrt{1-\eta^{2}}\theta_{t-1}^{G}\;,$$

where $v_{t}$ and $\theta_{t}$ are the current speed and direction of the target at time t; $\bar{v}$ and $\bar{\theta }$ are constants denoting the mean value of speed and direction; $v_{t-1}^{G}$ and $\theta_{t-1}^{G}$ are random variables from a Gaussian distribution, and η∈(0, 1) is a parameter that is used to vary the randomness of target motion. Therefore, the position of the target is calculated as follows:

(28) $$\left\{ \begin{array}{l} x_{t}=x_{t-1}+v_{t-1}\cos(\theta_{t-1})\;\;\\ y_{t}=y_{t-1}+v_{t-1}\sin(\theta_{t-1}) \end{array}\right.\;.$$

According to Equation (28), we can obtain the future position of the target in simulation. Not limited to this, different target motion models and prediction methods can be used in practical applications.

### 5.2. Performance Analysis

**Figure 9:**
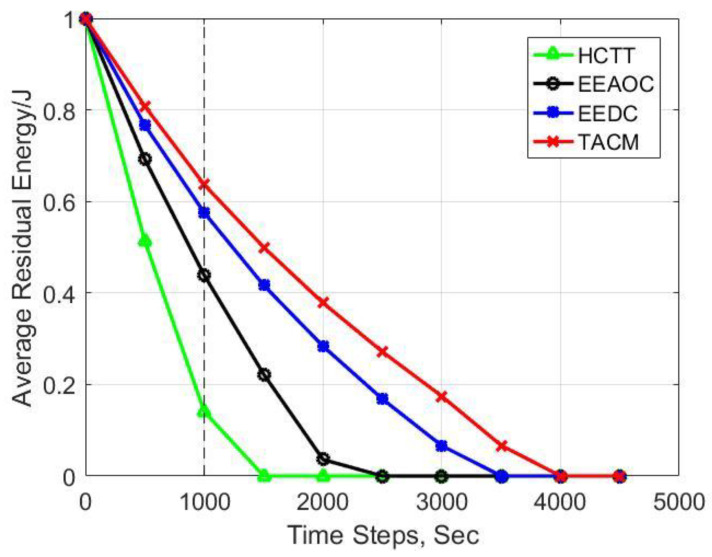
Experimental results for average residual energy of sensor nodes.

**Figure 10:**
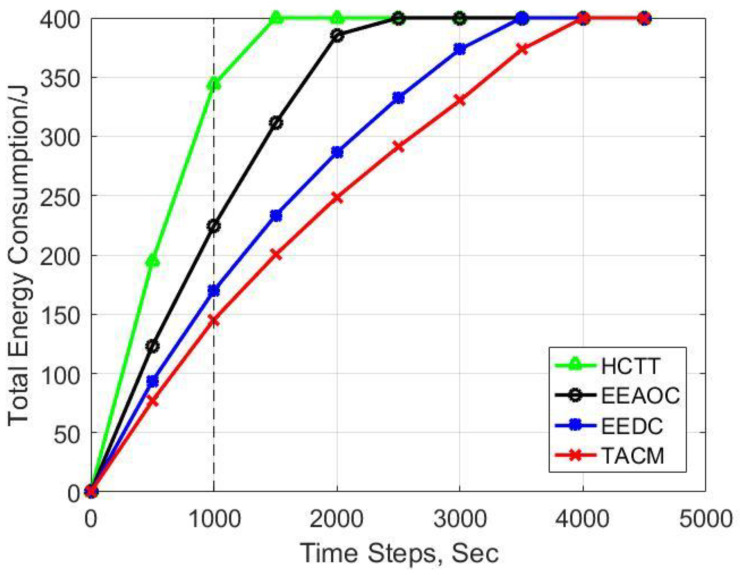
Experimental results for total energy consumption in the network.

We consider multiple vital metrics to measure the energy efficiency of different clustering methods for target tracking. First, we compared the residual energy of sensor nodes, and the average results of this metric are shown in Figure 9. Moreover, Figure 10 presents the total energy consumption during network operation. It can be seen that our proposed clustering method TACM displays better results in both metrics compared with other methods. At the 1000-th time step, the average residual energy is 0.64 joules for TACM, 0.57 joules for EEDC, 0.43 joules for EEAOC, and 0.14 joules for HCTT. Moreover, the total energy consumption is 145 joules for TACM, 169 joules for EEDC, 224 joules for EEAOC, and 343 joules for HCTT. The energy consumption of TACM is the lowest, which is achieved by introducing the tracking anchors to liberate nodes from the heavy workload of serving as fixed CHs. HCTT and EEAOC make structural adjustments on the static cluster as the target moves, so the communication overhead caused by CH rotation cannot be avoided. Although EEDC does not require static clusters as support, the selection of candidate CHs in an omnidirectional manner will result in energy waste, and the results show that its energy consumption is higher than that of TACM.

**Figure 11:**
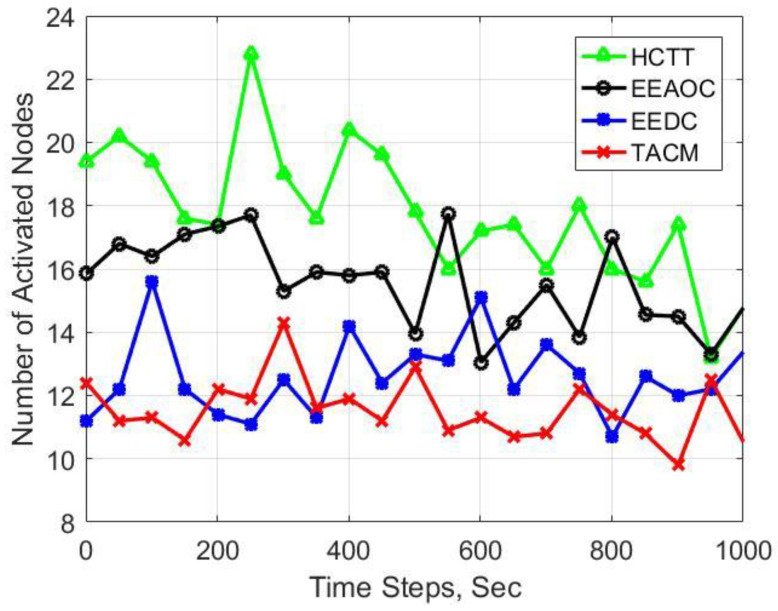
Experimental result for number of activated nodes in the network.

In Figure 11, the number of activated sensor nodes in the network is presented, and Table 2 gives the average values in 1000 time steps. It can be seen that the clustering methods that do not count on static cluster structure, such as EEDC and the proposed TACM, need to activate fewer sensors than HCTT and EEAOC in the process of target tracking. The reason is that, in HCTT, all the nodes in the nearby static cluster need to remain active to establish an on-demand cluster as CHs instructed while the target moves across clusters. In EEAOC, the logical overlapping cluster needs to be switched when the target crosses clusters, so the node is activated to interact. Moreover, compared with TACM, more nodes are activated in EEDC because its introduced candidate CHs need to be activated on standby for tracking tasks.

**Figure 12:**
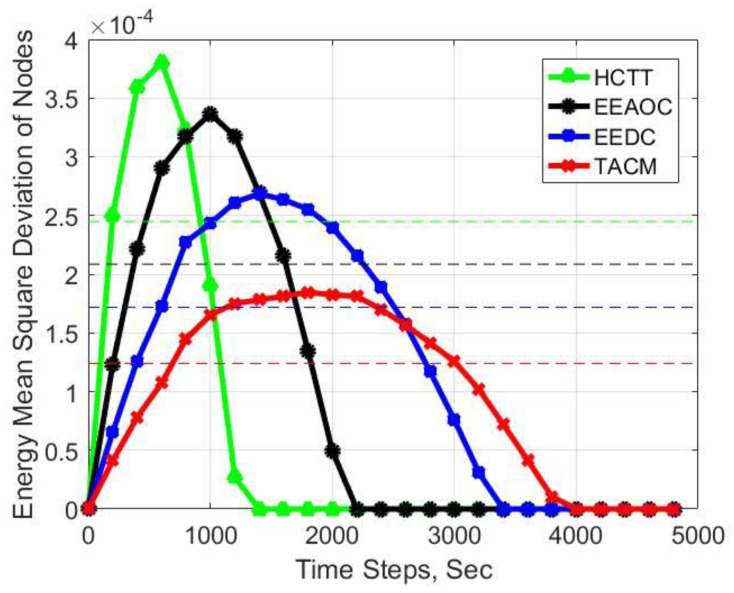
Experimental result for number of energy mean square deviation of nodes.

We calculate the energy mean square deviation of the living nodes to measure the energy distribution in the network, and the results are shown in Figure 12. The energy mean square deviation is $2.4483\times 10^{-4}$ for TACM, $2.085\times 10^{-4}$ for EEAOC, $1.7181\times 10^{-4}$ for EEDC, and $1.2395\times 10^{-4}$ for HCTT. It can be seen that the slope of TACM is smaller than that of the other three methods, indicating that the load balancing performance of TACM is the best. The reason is that the tracking anchors in TACM are only responsible for node activation, not the task of sending and receiving data, so they have a light workload. Moreover, CH is temporarily selected by volunteering according to the position of the target and the residual energy of the node so that the node serving as CH will not consume too much energy. In comparison, the CHs in EEAOC and HCTT cannot be changed in time like TACM, resulting in significant differences in energy consumption between nodes. Although the CH in EEDC is also temporarily selected, in the process of performing the candidate CHs selection process, the original CH undertakes additional information collection and calculation tasks, so the node serving as the CH will have a heavier burden than TACM.

**Figure 13:**
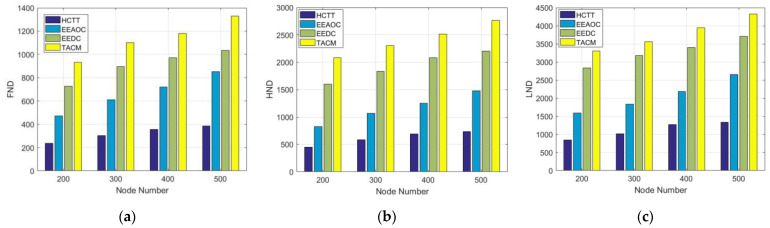
Experimental result for network lifetime (**a**) First node dead; (**b**) Half node dead; (**c**) Last node dead.

At last, we use the network lifetime to make an overall measure of the energy efficiency of the four clustering methods. Figure 13 shows the network lifetime with different numbers of nodes, and (a), (b), and (c) are the simulation results measured based on metrics called First Node Dies (FND), Half of the Nodes Die (HND), and Last Node Dies (LND). It can be seen that the proposed TACM method shows the best performance in all three metrics. In addition to avoiding frequent CH rotation and balancing node workload, as mentioned earlier, TACM also schedules the transmission status of CMs in the cluster. Specifically, only a part of the nodes that meet the tracking requirements with a miniature sensing overlap area between each other can transmit data. In this way, the transmission of redundant data can be reduced, thereby further saving energy and prolonging the network lifetime.

### 5.3. Threats to Validity

In this subsection, we discuss some threats to the validity of our conclusions. In the surveillance area of the network, we use the RFCM algorithm to determine the position of tracking anchors and the membership of nodes. Based on an ideal assumption, the anchors can be placed directly at the calculated position using transport, which is environmentally demanding and requires additional resources. Therefore, in the experiments, we designate the sensor node closest to the calculated position to work as the tracking anchor. However, due to slight changes in anchor positions, some nodes will be classified in the wrong positions, which leads to the risk of unreasonable clustering or even isolated nodes in the network. In future work, we will continue to improve this work to compensate for the performance loss due to anchor position bias.

## 6. Conclusions

Aimed at the tracking requirements for the moving target in WSNs, we propose a novel clustering method named TACM in this paper. Abandoning the static clusters formed by selecting CHs in the surveillance area, we introduce tracking anchors as indicators of sensor activation. Tracking anchors do not undertake the work of data transceiver and fusion so that they can avoid the overhead caused by frequent rotation. We utilize the RFCM algorithm to determine the location of the anchors and then build a membership table based on whether the sensor is located in the lower approximation region or boundary region. After that, sensors are activated to form a cluster according to activation rules and the membership table. The activated sensors will voluntarily become the CH using a distance and energy related delay broadcast. Since the CH in TACM is determined temporarily, there is no overload problem that exists with static CHs, and the load balance can be guaranteed. Furthermore, we set sensing and sensing-transmitting states for CMs in the cluster. Using linear 0–1 programming, we schedule the optimal work states for CMs. On the premise of meeting the tracking requirement, the CMs in the sensing state do not need to send data to the CH. In this way, the energy waste caused by redundant transmission can be reduced. The proposed method is compared with some fundamental and state-of-the-art existing clustering methods, and the simulation results indicate that TACM can achieve a more efficient data transmission process and can significantly reduce the overall energy consumption and prolong the network lifetime.

## Figures and Tables

**Table 1:** Simulation parameters.

Parameters	Value
Network Scale ($m^{2}$)	500 × 500
Node Number	200–500
Sink Coordinates (m)	(250, 250)
Initial Energy (J)	1
$E_{elec}$ (nJ/bit)	50
$\varepsilon_{fs}$$\;(pJ/bit/m^{2}$)	10
$\varepsilon_{mp}$$\;(pJ/bit/m^{4}$)	0.0013
Data Packet Size (bit)	4000
Transmission Round (s)	0.5
Sensing Radius $R_{s}$ (m)	20
Communication Radius $R_{c}$ (m)	40
Maximum Iteration $I_{\max}$	300
Change Rate Threshold ε	0.01

**Table 2:** Average number of activated nodes in 1000 time steps.

Clustering Methods	Average Number of Activated Nodes
HCTT	21.3
EEAOC	15.6
EEDC	12.3
TACM	10.5
